# Essential Oil of *Myrtus communis* L. as a Potential Antioxidant and Antimutagenic Agents

**DOI:** 10.3390/molecules15042759

**Published:** 2010-04-15

**Authors:** Neda Mimica-Dukić, Dušan Bugarin, Slavenko Grbović, Dragana Mitić-Ćulafić, Branka Vuković-Gačić, Dejan Orčić, Emilija Jovin, Maria Couladis

**Affiliations:** 1 Department of Chemistry, Biochemistry and Environmental Protection, University of Novi Sad Faculty of Sciences. Trg Dositeja Obradovica 3. 21 000 Novi Sad, Serbia; 2 Chair of Microbiology, Faculty of Biology, University of Belgrade. Studentski Trg 3/II, 11 000 Belgrade, Serbia; 3 Deparatment of Pharmacognosy and Chemistry of Natural Products, University of Athens, Panepistimioupolis, Zografou 157 71, Athens, Greece

**Keywords:** *Myrtus communis* L., essential oil, α-pinene, 1,8-cineole, myrtenyl acetate, mutagenesis, DPPH test

## Abstract

The present study describes DPPH (2,2-diphenyl-1-picrylhydrazyl) radical scavenging activity and antimutagenic properties of the essential oil of myrtle (*Myrtus communis* L.). Plant samples were collected from the two distant localities (southernmost and northern point) of the Montenegro coastline. Chemical profiles of the two samples were evaluated by GC-MS. In both of the samples monoterpenes were found to be the predominant compounds. Among them α-pinene, linalool, 1,8-cineole, and myrtenyl acetate were the major compounds. Significant differences between the samples were found in the ranges of α-pinene (14.7%–35.9%) and myrtenyl acetate (5.4%–21.6%). Both oils exhibited moderate DPPH scavenging activity, with IC_50_ values of 6.24 mg/mL and 5.99 mg/mL. The antimutagenic properties were assayed against spontaneous and *t*-BOOH-induced mutagenesis in *Escherichia coli oxyR* mutant IC202, a bacterial strain deficient in removing ROS. Reduction of the spontaneous mutagenesis in presence of myrtle *EO* was only slight, up to 13% at the highest concentration tested. When the oxidative mutagen was used, *EO* expressed higher reduction of mutagenesis, in a concentration dependent manner, with statistical significance for effect at the highest concentration tested (28%). Suppression of *t*-BOOH induced mutagenesis was correlated with the observed scavenging activity.

## 1. Introduction

There is increasing evidence that oxidative stress, defined as an imbalance between levels of various oxidant molecules and antioxidants, leads to many biochemical changes and consequently serious disorders in the human organism. Oxidative stress can result in damage to basic biomolecules, such as lipids, proteins and DNA, leading to cytotoxic and genotoxic effects [1,2]. Today, it is evident that mutagenicity and other adverse effects of reactive oxygen species (ROS) are implicated in aging, atherosclerosis, cancer, diabetes, and several neurodegenerative diseases [3,4,5,6]. Induction of mutagenesis occurs mainly through the damage of DNA by the HO^.^ radical and other species produced by radiolysis of water, and also by the direct effects of radiation on DNA. The reaction of most toxic hydroxyl HO^.^ radicals is mainly an addition to the double bonds of pyrimidine bases and abstraction of hydrogen from the sugar moiety resulting in DNA chain scission. Lesions to DNA resulting from attack by reactive oxygen species (ROS) can be a major cause of mutagenesis and carcinogenesis [7].

In the last two decades much emphasis has been placed on natural antioxidants. Therefore, both the naturally occurring nutritive and non/nutritive antioxidants have become a major area of scientific research [8,9]. The protection provided by fruits and vegetables against mutagenicity and cytotoxicity has been attributed to the various natural antioxidants they contain [10,11]. Among them phenolic substances such as tocopherols (vitamin E and related compounds), various classes of flavonoids, phenolic acid, tannins, lignans, *etc.*, are of special significance [12].

Spices and essential oils are also well known for their various beneficial effects on human health. The use of aromatic plants and spices in phytotherapy is mostly due to the essential oils and their various biological activities, such as antimicrobial, spasmolytic, carminative, hepatoprotective, antiviral, and anticarcinogenic properties [13,14]. Many essential oils and their constituents have recently been qualified as natural antioxidants and proposed as potential substitutes for the synthetic ones [15,16,17,18]. Due to their radical scavenging properties, antioxidants are believed to be directly antimutagenic and anticarcinogenic [14,19]. One of the mechanisms of their antimutagenic properties is inactivation of mutagens by the direct scavenging of reactive species (ROS) produced either by mutagens or by activation of cell antioxidant enzymes [14,20,21]. The role and reactions of ROS scavengers in preventing mutagenesis have been well documented in recent years. Berić *et al*., [22] and Mitić-Ćulafić *et al. *[23] have reported on the protective role of basil oil and some volatile monoterpenes against oxidative DNA injury and mutagenesis in bacterial strains sensitive to oxidative stress.

Myrtle (*Myrtus communis* L.) is an evergreen shrub belonging to the Myrtaceae family. It grows spontaneously throughout the Mediterranean area and has been used for medicinal, food and spice purposes since ancient times. The leaves and fruit are traditionally used as antiseptic, disinfectant, and hypoglycemic agents [24]. In folk medicine the fruit of the plant is used in the treatment of various infectious diseases, including diarrhea and dysentery, whereas the leaves are used as antiseptic and antiinflamatory agents, as a mouthwash, for treatments of candidiasis, for healing wounds, as well as in the therapy of urinary diseases [25,26].

A striking feature of the plant is the pleasant smell of its essential oil, present in numerous glands, especially in the leaves. The main compounds responsible for the flavor and scent of myrtle oil are monoterpenes: 1,8-cineole, myrtenyl acetate, α-pinene, myrtenol, limonene, *etc.* The oil composition is highly influnced by the geographic origin of the plant [27,28,29]. According to the numerous published papers on the topic, myrtle essential oil possesses strong antimicrobial activity that makes it a valuable raw material for the cosmetic, pharmaceutical and foodstuff industries [30,31].

Until now several studies have indicated that myrtle herbs could be used as a source of antioxidant and antimutagenic agents [19,32]. Generally, these studies were mainly focused towards the phenolic compounds in myrtle extracts [27]. Nevertheless, no reports on the comparative antioxidant and antimutagenic activity of myrtle essential oil have been available up to this moment. In the present study screening of antimutagenicity *via* the radical scavenging properties of myrtle (*M. communis*) essential oil (*EO*) from Montenegro has been continued. Radical scavenging activity was assyed by the DPPH-test, whereas antimutagenic potential was evaluated in the reverse mutation assays with *Esherichia coli* WP2 IC185 and its *oxyR* mutant IC202 strain that is deficient in the induction of antioxidant enzymes [33].

## 2. Results and Discussion

### 2.1. Essential Oil Composition

The oil yields in the examined myrtle leaves were 0.72 g/100 g dried leaves (*S1-*Ulcinj) and 0.81 g/100 g dried leaves (*S2-*Herceg Novi), respectively. If compared with oil yields of plants originating from other Mediterranean countries [27,28,29], like Greece and Spain (typically 0.4%–0.5%), myrtle plants from Montenegro could be characterized as being very rich in essential oils.

Twenty nine constituents, which represent 98.3% (*S1*) and 98.5% (*S2*) of the total *EO*, were identified. In both oil samples the major compounds belonged to the oxygenated monoterpene class, that accounted for 49.5% (*S2*) up to 70.1% (*S1*). In both oils α-pinene (14.7% *S1;* 35.9% *S2*), 1,8-cineole (25.7% *S1*; 23.9% *S2*), linalool ( 10.1% *S1*; 10.9% *S2*) and myrtenyl acetate (5.4% *S2*; 21.6% *S1*) were the dominant compounds (Table 1). According to Bradesi *et al*. [28], myrtle plants from Montenegro could be classified in the group with considerable and detectable amounts of myrtenyl acetate. The two examined samples could be further divided into one with a high content of α-pinene and a low content of myrtenyl acetate (*S2*), and the other with high myrtenyl acetate and low α-pinene (*S1*). Taking into account that plants were collected from the two distant localities (sothernmost and a northern point) of the Montenegro coastline, differences in oil composition are most probably a consequence of the environmental factors. According to the ratio of other compounds (linalool, 1,8-cineole) we found our samples similar to those reported from Greece [27], with the exceptions of considerable amounts of limonene found in our samples.

### 2.2. Antioxidant Activity

The results from the DPPH test for myrtle *EO* are presented in Table 2. Assessed essential oils were able to reduce the stable radical DPPH to the yellow-colored DPPH-H reaching 50% of reduction with an IC_50_ of 6.24 μL/mL and 5.99 μL/mL, respectively. The radical scavenging capacity of myrtle *EO* were significantly lower compared to commercial antioxidants (BHT, BHA and PG) examined in this study. This result is in agreement with the previously published data in which myrtle oil was less effective compared to the peppermint oil [31,18]. Evidently, no difference in DPPH-RSC between examined samples was found. Taking into account that *EO* samples differed mostly in the content of α-pinene and myrtenyl acetate, it is reasonable to assume that these two compounds are not responsible for radical scavenging activity. This assumption is further proved by the dot-blot test. The identification of the constituents most responsible for RSC was accomplished by comparing the control TLC analysis with the results of GC-MS (Table 1) and the TLC-DPPH method (Table 3).

In the essential oil, only 1,8-cineole and methyl eugenol showed considerable DPPH scavenging activity. Both compounds were previously reported as potent radical scavengers, especially methyl eugenol, most probably because of its phenylpropanoid moiety [17,34]. Presented results are in agreement with several studies of antioxidant abilities of volatile compounds, which proved that the highest antioxidant ability is shown by the compounds with phenolic or aromatic moieties in their molecule structures [18,34]. Although myrtle oils did not exhibit very strong DPPH scavenging activity, especially compared to synthetic antioxidants, this does not exclude myrtle oil as a potential natural antioxidant. It has to be kept in mind that only one test is not enough to valorize antioxidant efficiency of plant products, especially due to their complex composition [35].

### 2.3. Antimutagenic Activity

The antimutagenic effect of the myrtle oil against spontaneous and *t*-BOOH-induced mutagenesis was tested in *E. coli* IC202, a bacterial strain deficient in removing ROS. Since the strain carries the *trpE65* mutation, we followed the effect by monitoring the percentage of Trp^+^ reversions. Deficiency in removing ROS is a consequence of mutation in *oxyR* gene, e.g., a lack in the OxyR function. The OxyR protein is a redox-sensitive transcriptional activator of genes encoding antioxidant enzymes: catalase-hydroperoxidase I, alkyl hydroperoxide reductase and glutathione reductase, which cells produce as a response to an oxidative stress [36]. Due to the *oxyR* mutation, the IC202 strain is highly sensitive to oxidative DNA molecule damage.

The results of antimutagenicity testing are shown in Table 4. Reduction of the spontaneous mutagenesis in presence of *M. communis* essential oil was only slight, up to 13% at the highest concentration tested. When the oxidative mutagen was used, *EO* displayed a higher reduction of mutagenesis, in a concentration dependent manner, with statistical significance for the effect at the highest concentration tested (28%). Recently Mitić-Ćulafic *et al*. [23] reported the protective activity of volatile monoterpenes (linalool, myrcene and 1,8-cineole) against the oxidant induced genotoxicity in bacterial cells (*Escherichia coli* WP2 IC185 strain and its *oxyR* mutant IC202) and cultured human cells (NC-NC and HepG2). They found that myrcene and linalool strongly suppressed *t*-BOOH induced mutagenesis. Furthermore, they reduced DNA damage, induced by *t*-BOOH*.* All these compounds are identified in myrtle oils, among which 1,8-cineole and linalool are present in substantial amounts. With regard to the ROS scavenging capacity, demonstrated by the DPPH/TLC dot-blot test, one can assume that 1,8-cineole is the species most responsible for the antioxidant activity, but at the same time, a synergistic effect with other components is of great importance for the overall activity of the oil. Bearing in mind that bacterial cells were treated with antioxidant (*EO*) and mutagen (*t*-BOOH) simultaneously, we can presume that reduction of mutagenesis is related to either scavenging of ROS or direct interaction with *t*-BOOH. Results obtained indicate that myrtle essential oil has a substantial protective activity against oxidant induced mutagenesis, which is predominantly mediated by their radical scavenging activity.

## 3. Experimental

### 3.1. Plant material and Chemicals

Plant material: The aerial part of the myrtle plants (*Myrtus communis* L.) were collected in August 2006 from the two distant region in Montenegro coastline: the district of Ulcinj, the southernmost point and the district of Herceg Novi, a northern point of the Montenegro seaboard. Voucher specimens were prepared and identified by Goran Anačkov, PhD, and deposited at the Herbarium of the Department of Biology and Ecology (No 2-1818; No 2-1823, BUNS Herbarium), University of Novi Sad Faculty of Sciences.

Chemicals: 1,1-Diphenyl-2-(2,4,6-trinitrophenyl)hydrazine (syn: 2,2-diphenyl-1-picrylhydrazyl, DPPH), 2-thiobarbituric acid (TBA), sulfanilamide, *tert*-butylated hydroxytoluene (BHT), were obtained from Fluka Chemie GmbH (Buchs, Switzerland). Trichloroacetic acid was purchased from Lach-Ner s.r.o. (Neratovice, Czech Republic), *n*-hexane (Merck, Darmstadt, Germany); propyl galate, butylated hydroxyanisole (BHA) were obtained by ICN Biochemical-Clevelend USA; *t*-butyl hydroperoxide (*t*-BOOH, Aldrich, CAS No. 75-91-2).

### 3.2. Essential Oil Isolation and Analysis

Air-dried plant materials were submitted to hydrodistillation according to Eur. Pharm. 4 [37], using *n*-hexane as a collecting solvent. The solvent was removed under vacuum, and the quantities of the essential oils were gravimetrically determined.

*GC-MS analysis of the essential oil*: Qualitative analysis of the essential oils was performed by gas chromatography-mass spectrometry (GC-MS). An Agilent Technologies 6890N-5975C system was used, with data acquisition parameters as follows: carrier gas - He, flow 1.0 mL/min, constant flow mode; injection volume 0.2 µL (split 50:1), inlet temperature 250 °C; Agilent Technologies HP-5MS 30 m × 0.25 mm × 0.25 µm column, temperature program: 50 °C for 1 min, 5 °C/min to 100 °C/min, 9 °C/min to 200 °C, hold 7.89 min; transfer line temperature 280 °C; electron ionization, electron energy 70 eV, Scan mode, mass range 35–400 Da, quadrupole temperature 150 °C, source temperature 230 °C. Acquired data were analyzed by Agilent Technologies MSD ChemStation software in conjunction with AMDIS (Automated Mass Spectral Deconvolution and Identification System) and NIST MS Search software. Two different mass spectra libraries were used for mass spectra identification: the Wiley Registry of Mass Spectral Data 7th Edition (338,000 spectra, 289,000 unique compounds) [38] and the NIST/EPA/NIH Mass Spectral Library 05 with 190,825 spectra and 163,198 unique compounds [39]. Identity was confirmed by Kovats retention indices comparison.

### 3.3. Antioxidant Activity Assay

*Free radical scavenging capacity (RSC):* The RSC was evaluated by measuring the scavenging activity of *EO* samples on the stable DPPH radical. The DPPH assay was performed as described before [34]. Various amount of essential oil samples (5, 10, 20, 40 and 60 mg) were mixed with 1 mL of 90 μM DPPH solution and made up to a final volume of 4 mL with 95% MeOH. The final concentrations of oils are reported in Table 2. In the control, the *EO* was substituted with a similar amount of solvent. The well known artificial synthetic antioxidants BHT, BHA and PG were used as a positive control. Monitoring was continued for 70 min until the reaction reached a plateau.

For each sample four replicates were recorded. The disappearance of DPPH was read spectrophotometrically at 515 nm using a Beckman DU-65 spectrophotometer. The RSC in percent was calculated by following equation:

RSC (%) = 100 − (A_blank_ − A_sample_/A_blank_)

From the obtained RSC values, the IC_50_ values, which represented the concentrations of the essential oils that caused 50% neutralization, were determined by linear regression analysis.

### 3.4. Rapid Screening for Scavenging Compounds of Essential Oils

For fast screening of essential oil compounds on RSC, the dot-blot test [40] on thin-layer chromatography (TLC) silica gel F_254_ aluminum plates (Merck) stained with the free radical DPPH was performed as previously described [34,41]. Shortly, an appropriate aliquot (5 µL) of *EO* was placed on the silica gel plates and chromatographed in the solvent system benzene-EtOAc (95:5). After the mobile phase had dried, the silica layer was stained by spraying with 0.4 mM solution of DPPH in MeOH using a Desaga Spray-Gun. The stained silica layer gave a purple background with yellow spots at the location of those compounds on TLC that possess high RSC. The control plate was developed simultaneously and under exactly the same conditions, and visualization of certain spots was done by spraying the developed plate with vanillin-sulfuric acid reagent. The most active RS compounds were identified by comparing the Rf values (Table 3) of the yellow spots with the corresponding ones on the control layer.

### 3.5. Antimutagenic Activity

*Bacterial Strains and Media:* The tester strains used in this study were *E. coli* WP2, strains IC185 *trpE65* and IC202 *trpE65 oxyR*/pKM101 [36]. *Media*: Bacteria were grown in (i) LB medium (5 g yeast-extract, 10 g bacto-tryptone, 5 g NaCl, 1000 mL distilled water; (ii) LA medium the same as LB but 15 g Difco agar was added; (iii) Solid minimal E4 medium containing 15 g Difco agar, 4 g glucose and 20 mL Vogel-Bonner E buffer; (iv) ET4 plates (minimal medium, supplemented with 0.5 mg/L tryptophan); (v) top agar containing 6 g Difco agar and 5 g NaCl (per liter of distilled water).

#### 3.5.1. Toxicity Assay

Toxicity of *EO* was determined by inhibition of bacterial growth of 0.1 mL overnight culture was added to 3 mL of molten top agar held at 45 °C and poured on LA plates. Different dilution of *EO* was added on sterile filter paper disc (6 mm in diameter), placed onto inoculated surface and incubated for 24 h at 37 °C. A clear zone of growth inhibition around the disc indicates a cytotoxic effect.

#### 3.5.2. Bacterial WP2 Antimutagenicity Assay

The overnight culture of *E. coli* IC202 was grown in LB medium at 37 °C. The antimutagenicity assay was performed by mixing 0.1 mL of fresh overnight cultures of bacteria, 0.1 mL of buffer dilution of *t*-BOOH (final concentration 25 μg/plate), appropriate concentration of *EO* (0.05, 0.75, 0.1 and 0.15 μL/plate) diluted in hexane, and 3 mL of molten top agar (45 °C). The mixture was poured onto minimal ET4 plates [36]. The number of Trp^+^revertants was scored after incubation for 48 h at 37 °C. Simultaneously, the influence of *EO* on spontaneous mutagenesis was examined. Plating on E4 (minimal medium, without tryptophan) was used to evaluate the number of pre-plating mutants originating during the overnight growth. The overnight cultures with a low number of preexisting revertants (<15 revertants per plate) were used for experiments. Moreover, the potential toxic effect of *EO* on viability of cells untreated and treated with *t*-BOOH and by plating appropriate diluted overnight cultures on LA plates we tested. Hexane was used as a negative control. Experiments were carried out at least 3 times.

### 3.6. Statistical Analysis

The t-test was employed for statistical analysis. The significance was tested at the p < 0.05 level. Values in Tables are averages with standard errors. In experiments we calculated the percentage of inhibition of mutagenesis (%I) as described by Wall *et al.* [42].

## 4. Conclusions

This paper describes a comparative study of the chemical composition, antioxidant and antimutagenic properties of the essential oil obtained from the leaves of wild growing *Myrtus communis* from the Montenegro coastline. In light of the results obtained we can conclude that myrtle essential oil showed considerable antimutagenic potential (in a non-toxic concentration range), which was probably due to the antioxidant activity of its components. Combined TLC and DPPH-tests show that 1,8-cineole and methyl eugenol are the compounds most responsible for the scavenging activity of the entire oil. According to some recent literature data [19,32,43] it is reasonable to assume that phenolic compounds present in the methanol or ethanol extracts of myrtle leaves, will additionally contribute to the overall antioxidant and antimutagenic potential. To conclude, myrtle plants could be a promising source of natural antioxidants, anti-genotoxic, antimutagenic and, perhaps, chemopreventive agents. Nevertheless, additional *in vitro* and *in vivo* studies are needed to unequivocally demonstrate this.

## Figures and Tables

**Table 1:** Chemical composition (%) of *Myrtus communis* essential oil collected from two localities of the Montenegro coastline: Ulcinj (*S1*) and Herceg Novi (*S2*).

No.	Components	R.I.^a^	*S1*	*S2*
1	Isobutyl isobutyrate	914	1.9	0.7
2	α-Thujene	927	0.4	0.4
3	α-Pinene	934	**14.7**	**35.9**
4	β-Pinene	978	0.2	0.3
5	β-Myrcene	992	0.3	0.2
6	α-Phellandrene	1006	0.4	0.3
7	δ-3-Carene	1012	0.5	0.4
8	α-Terpinene	1018	0.3	0.2
9	*p*-Cymene	1026	0.9	1.2
10	Limonene	1030	4.1	4.5
11	1,8-cineole	1032	**25.7**	**23.9**
12	(*E*)-β-Ocimene	1049	0.4	0.3
13	γ-Terpinene	1060	0.9	0.7
14	α-Terpinolene	1090	1.1	0.8
15	Linalool	1101	**10.1**	**10.9**
16	4-Terpineol	1182	0.3	0.3
17	Cryptone	1191	tr^b^	0.2
18	α-Terpineol	1194	3.1	2.8
19	Myrtenol	1200	0.8	0.6
20	Geraniol	1256	2.6	1.6
21	Myrtenyl acetate	1325	**21.6**	**5.4**
22	α-Terpinyl acetate	1355	1.4	0.5
23	Neryl acetate	1367	0.3	0.2
24	Geranyl acetate	1385	3.4	2.3
25	Methyl eugenol	1406	0.8	1.0
26	(*E*)-β-Caryophyllene	1420	0.6	0.5
27	α-Humulene	1460	1.5	1.4
28	Bicyclogermacrene	1500	tr	0.2
29	Spathulenol	1593	tr	0.8
	Identified compounds		98.3	98.5
	Monoterpene hydrocarbons		24.2	45.3
	Oxygenated monoterpenes		**70.1**	49.5
	Sesquiterpene hydrocarbons		2.1	2.1
	Oxygenated sesquiterpenes		-	0.8

^a^ Retention indices relative to C_9_–C_24_ n-alkanes on the HP-5MS column; ^b^ tr-abundance in essential oil below 0.2%.

**Table 2:** DPPH-radical scavenging capacity (RSC) of *Myrtus communis* essential oils: *EOS1* and *EO-S2* and BHA, BHT and PG as positive controls.

*EO*	Concentration (μg /mL) × 10^3^	Inhibition (%)	IC_50_ (μg /mL) × 10^3^
*EO*-S1	1.25	17.50	**6.24**
	2.50	25.16	
	5.00	44.46	
	10.00	67.29	
	15.00	85.12	
*EO*-S2	1.25	25.50	**5.99**
	2.50	30.84	
	5.00	47.26	
	10.00	63.12	
	15.00	72.71	
Standard antioxidants			IC_50_ (μg/mL)
BHT			**8.62**
BHA			**3.09**
PG			**0.42**

**Table 3:** DPPH Scavenging active compounds identified by the means of DPPH/ TLC (dot-blot) technique.

Spot No	Compound	Rf values	Positive reaction to DPPH *
1	α-Terpineol	0.26	
2	Linalool	0.42	
3	1,8-Cineole	0.55	+
4	Methyl eugenol	0.64	+
5	Mixture of acetylated monotepenoids	0.76	
	alcohols (myrtenyl acetate, linalyl		
	acetate, geranyl acetate)		
6	α-pinene	0.95	

* Yellow spot appearance.

**Table 4:** Effect of *Myrtus communis* essential oil on spontaneous and *t*-BOOH-induced mutagenesis in IC202.

Concentration (μL/plate)	- t-BOOH		+ t-BOOH^1^	
	revert/pl.^2^	%I^3^	revert/pl.	%I
0				
n-hexane	97 ± 16	100	196 ± 13	100
0.05	92 ± 17	94	187 ± 17*	95
0.075	101 ± 20	104	159 ± 7	81
0.1	88 ± 15	90	163 ± 22	83
0.15	85 ± 14	87	142 ± 17	72

^1^ *t*-BOOH induced mutagenesis, applied concentration 25μg/pl. The presented values are average of duplicate samples from three different experiments; ^2^ Trp/mL = Trp^+^ revertants/plx10; ^3^ %I = (1-N_t_/N_c_) × 100. N_t_, sample with *EO*; N_c_ control sample (*n*-hexane); ** p≤ 0.05; * statistically significant values.
